# Society Meetings

**Published:** 1896-06

**Authors:** 


### Society Meetings.

The Second Pan-American Medical Congress.—The committee on organisation of the Second Pan-American Medical Congress has elected Dr. Manuel Carmona y Valle, president, Dr. Rafael Lavista, vice-president, and Dr. Eduardo Liceaga, secretary, and has announced November 16, 17, 18, 19, 1896, as the date of the meeting to be held in the city of Mexico.

The most cordial invitation is extended to the medical profession of the United States to attend and participate in the meeting.

Titles of papers to be read should be sent at the earliest practicable date to Dr. Eduardo Liceaga, Calle de San Andres num. 4, Ciudad de Mexico D. F. Republica Mexicana.

The date selected will find Mexico most attractive to a Northern visitor.

The occasion should stimulate the medical profession of the United States to a most cordial reciprocation of the generous patronage accorded the Washington meeting of the congress by our Mexican confréres. It should be remembered that the United States is the largest, and in many regards the most important of the American countries, and that as a consequence more is expected of it than of any other occidental nation. It is, therefore, simply essential that in this congress—the most important of all medical congresses, in its exclusive, yet broad, American significance—the best thought and the best work of the American profession shall be conspicuous in the proceedings.

Those who contemplate attending should send their names and addresses at as early a date as possible to Dr. Charles A. L. Reed, St. Leger Place, Cincinnati, that the committee in Mexico may be advised of the probable attendance.

WILLIAM PEPPER,
*ex-officio President.*
A. M. OWEN,
A. VANDER VEER,
CHARLES A. L. REED,
*ex-officio Secretary.*
*International Executive Committee for the United States.*

---

THE National Confederation of State Medical Examining and Licensing Boards held its sixth annual meeting at Atlanta, Ga., May 4, 1896. The following officers were elected for the ensuing year : president, William Warren Potter, M. D., Buffalo, N. Y. ; vice-presidents, Charles A. L. Reed, M. D., Cincinnati, Ohio, and J. N. McCormick, M. D., Bowling Green, Ky. ; secretary and treasurer, A. Walter Suiter, M. D., Herkimer, N. Y. ; executive council, Perry H. Millard, M. D., St. Paul, Minn. ; Joseph M. Mathews, M. D., Louisville, Ky. ; William S. Foster, M. D., Pittsburgh, Pa. ; Hugh M. Taylor, M. D., Richmond, Va., and James Mackintosh Hays, M. D., Greensboro, N. C.

The president appointed the following committee on the minimum standard of requirements : Drs. Perry H. Millard, Minn., chairman ; N. R. Coleman, Ohio ; B. M. Griffith, Illinois ; J. M. Hays, North Carolina, and Gardiner T. Schwartz, Rhode Island.

---

THE American Medical Association held its forty-ninth annual meeting at Atlanta, Ga., May 5, 6, 7 and 8, 1896. The following officers were elected : president, Dr. Nicholas Senn, Illinois ; first vice-president, Dr. George M. Sternberg, Washington, D. C.; second vice-president, Dr. Edmond Souchon, Louisiana ; third vice-president, Dr. J. B. Thomas, Pennsylvania ; fourth vice-president,

Dr. Willis F. Westmoreland, Georgia; treasurer, Dr. Henry P. Newman, Illinois; assistant secretary, Dr. T. B. Schneideman, Pennsylvania; librarian, Dr. George W. Webster, Illinois; chairman of committee of arrangements, Dr. H. A. Hare, Pennsylvania; trustee to fill vacancy, Dr. G. C. Savage, Tennessee; trustees, Dr. E. E. Montgomery, Pennsylvania; Dr. J. M. Mathews, Kentucky, and Dr. C. A. L. Reed, Ohio; judicial council, Dr. George W. Stoner, U. S. Marine Hospital service; Dr. C. W. Foster, Maine; Dr. J. McFadden Gaston, Georgia; Dr. I. M. Quimby, New Jersey; Dr. H. Brown, Kentucky, and Dr. X. C. Scott, Ohio. Address in surgery, Dr. W. W. Keen, Pennsylvania; address in medicine, Dr. Austin Flint, New York; address in state medicine, Dr. J. Cochran, Alabama. The semi-centennial meeting of the Association will be held in Philadelphia, June, 1897.

---

The American Orthopedic Association held its tenth annual meeting in Buffalo, May 19, 20, 21, 1896. The meeting came too late for anything more than the briefest notice in the Journal. The opening session was held at Alumni hall, University building, High street, at which time the president, Dr. Royal Whitman, of New York, delivered his address on The scope of orthopedic surgery. Dr. Bernard Bartow, of Buffalo, read a paper before the society on Division of the hamstring tendons by the open method for correcting the malposition and securing rest in tubercular disease of the knee. Officers elected were: president, Dr. Samuel Ketch, New York; 1st vice-president, Dr. Henry M. Sherman, San Francisco; 2d vice-president, Dr. L. A. Weigel, Rochester; treasurer, Dr. E. G. Brackett, Boston; secretary, Dr. John Ridlon, Chicago. Dr. Bernard Bartow and Dr. Roswell Park were the local committee of arrangements.

---

The twelfth annual meeting of the New York State Medical Association (Fourth District branch) was held May 12, 1896, at the parlors of the Iroquois hotel, Buffalo, N. Y. The president, Darwin Colvin, M. D., being absent, Dr. Charles G. Stockton presided. The following papers were read: A recent experience with erythema nodosum trachialis, George F. Cott, M. D.; Reports of especially interesting cases in abdominal surgery, C. C. Frederick, M. D.; Acute catarrhal gastritis, Charles G. Stockton, M. D.; Two cases of intrathoracic growths, De Lancey Rochester, M. D.

The American Public Health Association will hold its annual meeting at Buffalo, September 15-18, 1896. The chairman of the local committee of arrangements, Dr. Ernest Wende, with the energy that characterises all his undertakings, is laying plans for a hearty welcome to the distinguished savants whom this meeting will call together. The gathering bids fair to be most successful and profitable.

---

The fiftieth annual session of the Wisconsin State Medical Society will be held in the assembly chamber, Madison, Wis., June 3, 4 and 5, 1896. The preliminary program is attractive both in appearance and contents.

---

The Medical Society of the County of Erie will hold its regular semi-annual meeting at the Buffalo Academy of Medicine parlors, Tuesday, June 9, 1896, under the presidency of Dr. J. G. Thompson, of Angola.

---

The American Gynecological Society met in New York, May 26, 27 and 28, 1896.

